# Supplementary material for: Two-Dimensional NMR Lineshape Analysis
Source: Sci Rep. 2016 Apr 25;6:24826. doi: 10.1038/srep24826 (PMC4843008; doi:10.1038/srep24826)
Supplement: Supplementary Information [file srep24826-s1.pdf]

Two-Dimensional NMR Lineshape Analysis

Christopher A. Waudby[1,*], Andres Ramos[1], Lisa D. Cabrita[1], John Christodoulou[1,*]

[1] Institute of Structural and Molecular Biology, University College London and Birkbeck College, London, UK

[*] E-mail: c.waudby@ucl.ac.uk, j.christodoulou@ucl.ac.uk

SUPPLEMENTARY INFORMATION

Table S1 Superoperators for calculation of evolution during pulse sequences.

Figure S1 Comparison of 1D and 2D lineshape fitting showing effect of differential relaxation.

Figure S2 Comparison of exchange lineshapes in HSQC and HMQC experiments.

Figure S3 Analysis of pulse sequences for lineshape analysis.

Figure S4 Fit results for the titration of FIR RRM1-RRM2 with FBP Nbox.

Figure S5 Fit results for the titration of FIR RRM1-RRM2 with FBP3 Nbox.

Figure S6 Fit results for the titration of FIR RRM1-RRM2 with oligonucleotides.

Figure S7 Simulation of binding mechanisms.

Figure S8 Fit results for the titration of Ca^{2+}-CaM with TFP.

Figure S9 Comparison of exchange lineshapes in HSQC and CPMG-HSQC experiments.

	HSQC	HMQC	CPMG-HSQC
ρ_0	$p_0 \otimes (0\quad 1\quad 0\quad 0)'$	$p_0 \otimes (0\quad 1\quad 0\quad 0)'$	$p_0 \otimes (0\quad -1\quad 0\quad 0)'$
basis A	$\{I_x, I_y, 2I_xS_z, 2I_yS_z\}$	$\{I_x, I_y, 2I_xS_z, 2I_yS_z\}$	$\{I_x, I_y, 2I_xS_z, 2I_yS_z\}$
basis B	$\{S_x, S_y, 2I_zS_x, 2I_zS_y\}$	$\{ZQ_x, ZQ_y, DQ_x, DQ_y\}$	$\{S_x, S_y, 2I_zS_x, 2I_zS_y\}$
$L_{free,A}$	$\bigoplus_{i=1}^{K}\begin{pmatrix} -R^I_{2,i} & -\omega^I_i & 0 & -\pi J \\ \omega^I_i & -R^I_{2,i} & \pi J & 0 \\ 0 & -\pi J & -R^I_{2,i} & -\omega^I_i \\ \pi J & 0 & \omega^I_i & -R^I_{2,i} \end{pmatrix} + K \otimes I_4$	$\bigoplus_{i=1}^{K}\begin{pmatrix} -R^I_{2,i} & -\omega^I_i & 0 & -\pi J \\ \omega^I_i & -R^I_{2,i} & \pi J & 0 \\ 0 & -\pi J & -R^I_{2,i} & -\omega^I_i \\ \pi J & 0 & \omega^I_i & -R^I_{2,i} \end{pmatrix} + K \otimes I_4$	$\bigoplus_{i=1}^{K}\begin{pmatrix} -R^I_{2,i} & -\omega^I_i & 0 & -\pi J \\ \omega^I_i & -R^I_{2,i} & \pi J & 0 \\ 0 & -\pi J & -R^I_{2,i} & -\omega^I_i \\ \pi J & 0 & \omega^I_i & -R^I_{2,i} \end{pmatrix} + K \otimes I_4$
$L_{free,B}$	$\bigoplus_{i=1}^{K}\begin{pmatrix} -R^S_{2,i} & -\omega^S_i & 0 & -\pi J \\ \omega^S_i & -R^S_{2,i} & \pi J & 0 \\ 0 & -\pi J & -R^S_{2,i} & -\omega^S_i \\ \pi J & 0 & \omega^S_i & -R^S_{2,i} \end{pmatrix} + K \otimes I_4$	$\bigoplus_{i=1}^{K}\begin{pmatrix} -R^{MQ}_{2,i} & \omega^I_i - \omega^S_i & 0 & 0 \\ -\omega^I_i + \omega^S_i & -R^{MQ}_{2,i} & 0 & 0 \\ 0 & 0 & -R^{MQ}_{2,i} & \omega^I_i + \omega^S_i \\ 0 & 0 & -\omega^I_i - \omega^S_i & -R^{MQ}_{2,i} \end{pmatrix} + K \otimes I_4$	$\bigoplus_{i=1}^{K}\begin{pmatrix} -R^S_{2,i} & -\omega^S_i & 0 & -\pi J \\ \omega^S_i & -R^S_{2,i} & \pi J & 0 \\ 0 & -\pi J & -R^S_{2,i} & -\omega^S_i \\ \pi J & 0 & \omega^S_i & -R^S_{2,i} \end{pmatrix} + K \otimes I_4$
$P^x_{180,A}$	$I_k \otimes diag(1, -1, -1, 1)$		$I_k \otimes diag(1, -1, -1, 1)$
$P^y_{180,A}$			$I_k \otimes diag(-1, 1, 1, -1)$
$P^x_{180,B}$	$I_k \otimes diag(1, 1, -1, -1)$	$I_k \otimes \begin{pmatrix} 0 & 0 & 1 & 0 \\ 0 & 0 & 0 & -1 \\ 1 & 0 & 0 & 0 \\ 0 & -1 & 0 & 0 \end{pmatrix}$	$I_k \otimes diag(1, 1, -1, -1)$
G_{INEPT}	$e^{L_{free,A}T} P^x_{180,A} e^{L_{free,A}T}$ $(T = 1/4J)$	$e^{L_{free,A}T}$ $(T = 1/2J)$	$\left[\left(G_xG_y\right)^2\left(G_yG_x\right)^2\right]^2$ $(G_x = e^{L_{free,A}T} P^x_{180,A} e^{L_{free,A}T},\ G_y = e^{L_{free,A}T} P^y_{180,A} e^{L_{free,A}T},$ $T = 1/32J)$
R_{AB}	$I_k \otimes diag(0, 0, 1, 0)$	$I_k \otimes \begin{pmatrix} 0 & 0 & 0 & -1/2 \\ 0 & 0 & 1/2 & 0 \\ 0 & 0 & 0 & 1/2 \\ 0 & 0 & -1/2 & 0 \end{pmatrix}$	$I_k \otimes diag(0, 0, 1, 0)$
R_{BA}	$I_k \otimes \begin{pmatrix} 0 & 0 & 0 & 0 \\ 0 & 0 & 0 & 0 \\ 0 & 0 & 0 & 0 \\ 0 & 0 & 1 & -i \end{pmatrix}$	$I_k \otimes \begin{pmatrix} 0 & 0 & 0 & 0 \\ 0 & 0 & 0 & 0 \\ -i & -1 & -i & 1 \\ 1 & -i & -1 & -i \end{pmatrix}$	$I_k \otimes \begin{pmatrix} 0 & 0 & 0 & 0 \\ 0 & 0 & 0 & 0 \\ 0 & 0 & 0 & 0 \\ 0 & 0 & 1 & -i \end{pmatrix}$
M_+	$I_k \otimes (1\quad 0\quad 0\quad 0)$	$I_k \otimes (0\quad 1\quad 0\quad 0)$	$I_k \otimes (1\quad 0\quad 0\quad 0)$
M_{obs}	$M_+ G_{INEPT} R_{BA} e^{\frac{L_{free,B}t_1}{2}} P_{180,B} e^{\frac{L_{free,B}t_1}{2}} R_{AB} G_{INEPT} \rho_0$	$M_+ G_{INEPT} R_{BA} e^{\frac{L_{free,B}t_1}{2}} P_{180,B} e^{\frac{L_{free,B}t_1}{2}} R_{AB} G_{INEPT} \rho_0$	$M_+ G_{INEPT} R_{BA} e^{\frac{L_{free,B}t_1}{2}} P_{180,B} e^{\frac{L_{free,B}t_1}{2}} R_{AB} G_{INEPT} \rho_0$

Table S1. Superoperators for calculation of evolution during HSQC, HMQC and CPMG-HSQC pulse sequences (as depicted schematically in Fig. S3). *K* is the exchange matrix, *k* is the number of states, and I_n is the *n* x *n* identity matrix.

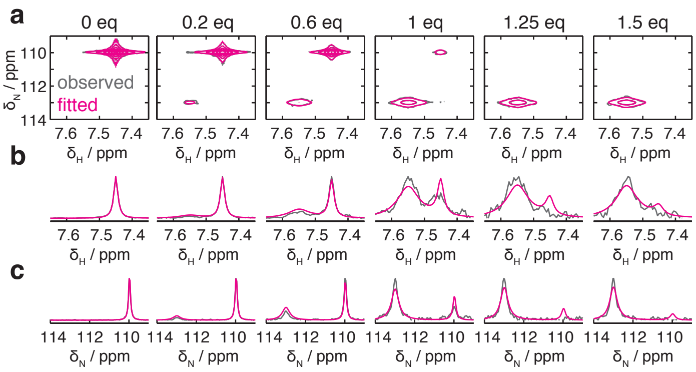

Figure S1. Comparison of one-dimensional and two-dimensional lineshape fitting showing effect of differential relaxation. (**a**) ^{1}H,^{15}N-HSQC titration data was simulated, including noise, for a two-state binding interaction (K_d 1 μM, k_{off} 5 s^{-1}) between two states with different relaxation rates (grey). The results of two-dimensional lineshape fitting (magenta) were in excellent agreement with the true parameters (best-fit K_d 1.02 μM, k_{off} 5.06 s^{-1}) (**b,c**) In contrast, simultaneous one-dimensional fits (magenta) to ^{1}H and ^{15}N cross-sections (grey) did not converge to the observed lineshapes (best-fit K_d 6.8 μM, k_{off} 5.5 s^{-1}).

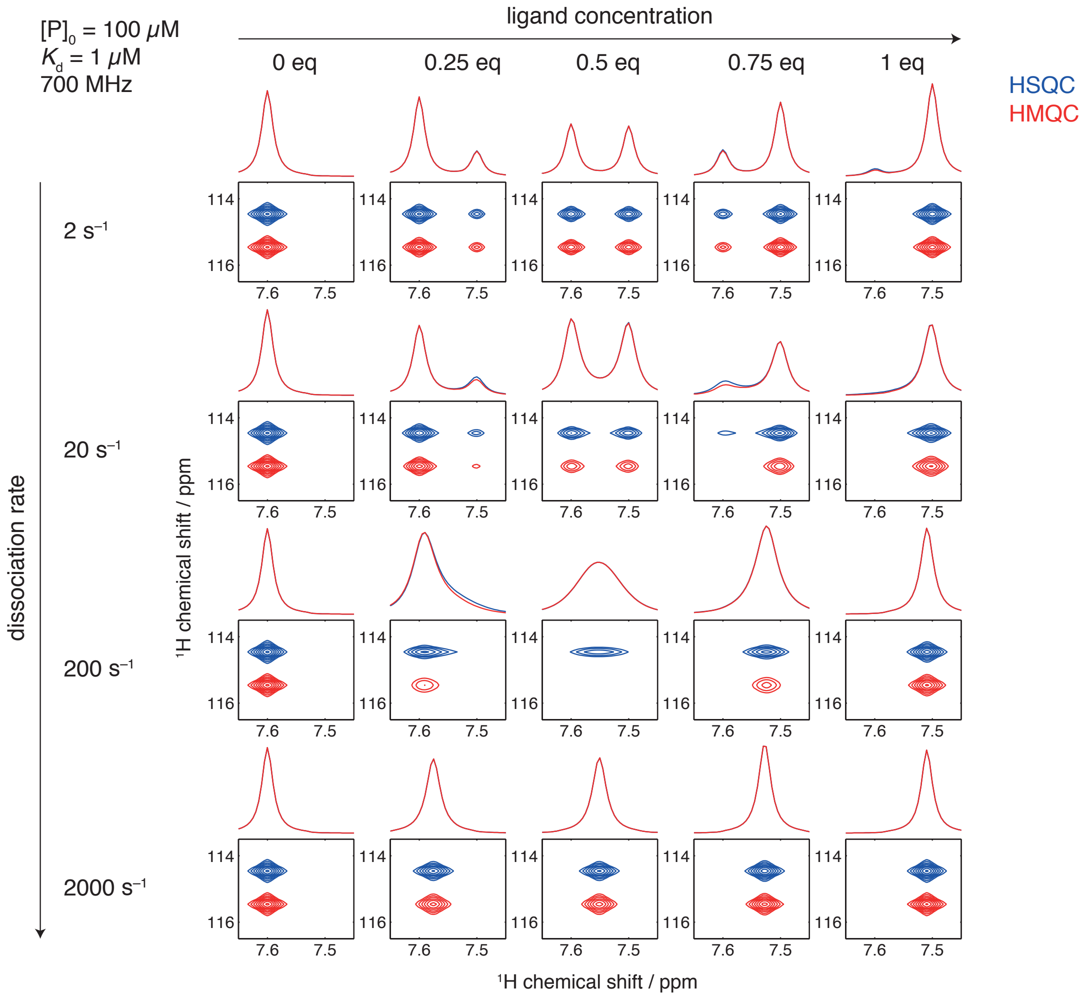

Figure S2. Comparison of simulated exchange lineshapes in $^{1}H,^{15}N$-HSQC and $^{1}H,^{15}N$-HMQC experiments for a titration of a protein with an interacting ligand (K_d = 1 μM, protein concentration $[P]_0$ = 100 μM, ^{1}H Larmor frequency = 700 MHz, and dissociation rates and ligand concentrations as indicated), illustrating the increased broadening in HMQC spectra due to exchange of multiple-quantum coherences during the indirect evolution period. Normalised cross-sections are also shown, highlighting the effect of differential broadening on lineshapes under conditions of asymmetric slow–intermediate exchange.

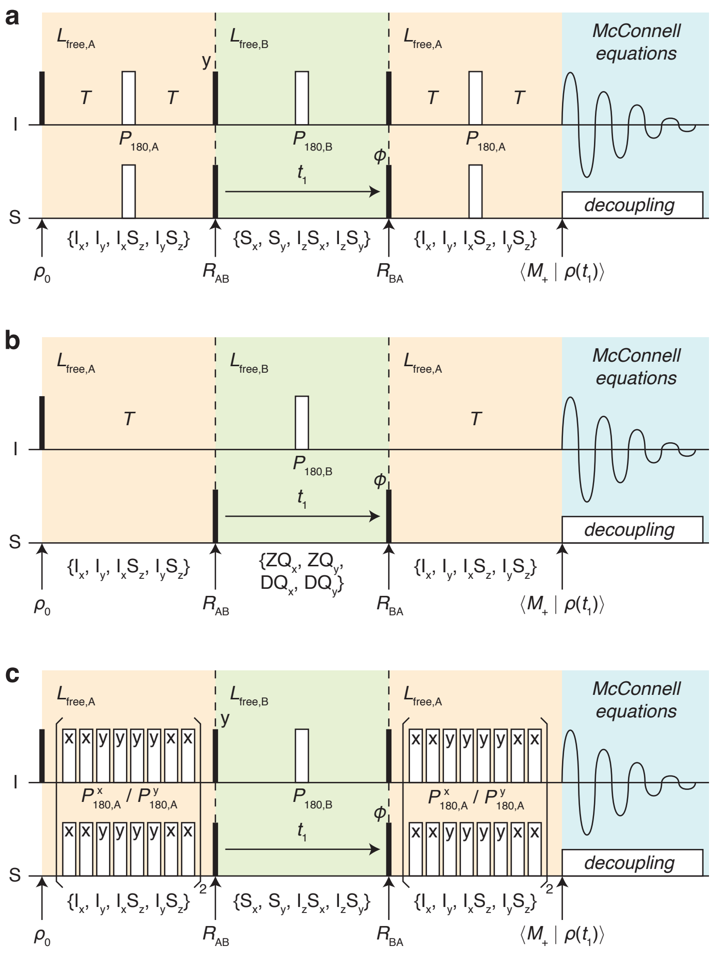

Figure S3. Schematic illustration of calculation schemes detailed in Table S1 for the efficient simulation of two-dimensional spectra obtaining using (**a**) HSQC, (**b**) HMQC, and (**c**) CPMG-HSQC pulse sequences. Solid bars indicate 90° pulses and hollow bars 180° pulses, with phase 'x' unless otherwise indicated. The initial density operator ρ_0 is propagated through the sequence in the reduced basis spaces indicated with braces and coloured shading, with the superoperators R indicating rotations between basis subspaces. The populations of in-phase proton magnetization are determined by projection (onto M_{+}) at the point of acquisition, with efficient calculation of lineshapes in the direct dimension subsequently obtained through solution of the classical Bloch-McConnell equations.

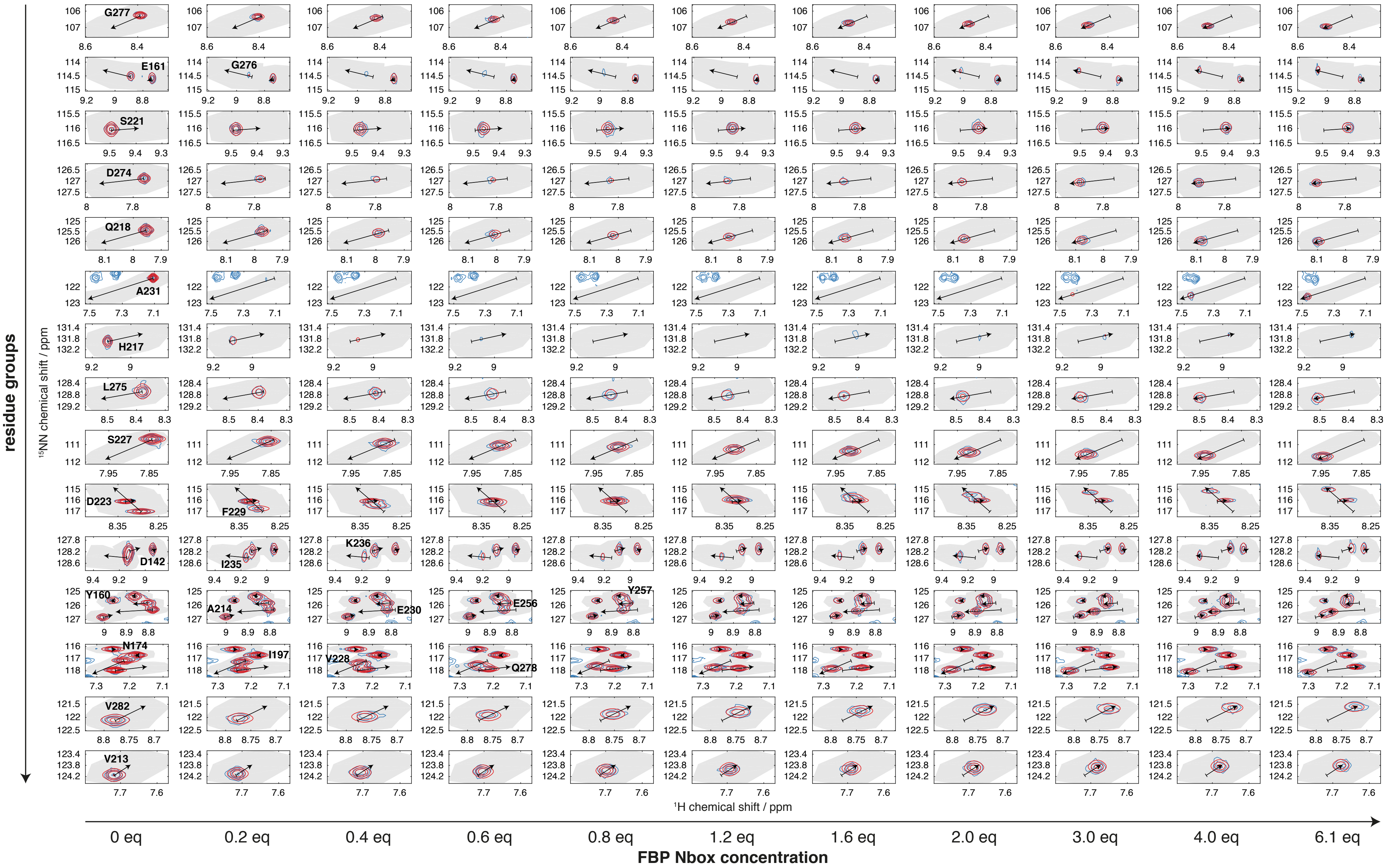

Figure S4. Results of two-dimensional lineshape analysis for the titration of FIR RRM1-RRM2 with FBP Nbox. Blue, observed; red, fitted. Shaded areas indicate the selected regions of interest (ROIs). The chemical shifts of free and bound states determined by the fitting procedure are marked by the tail and head of the arrows.

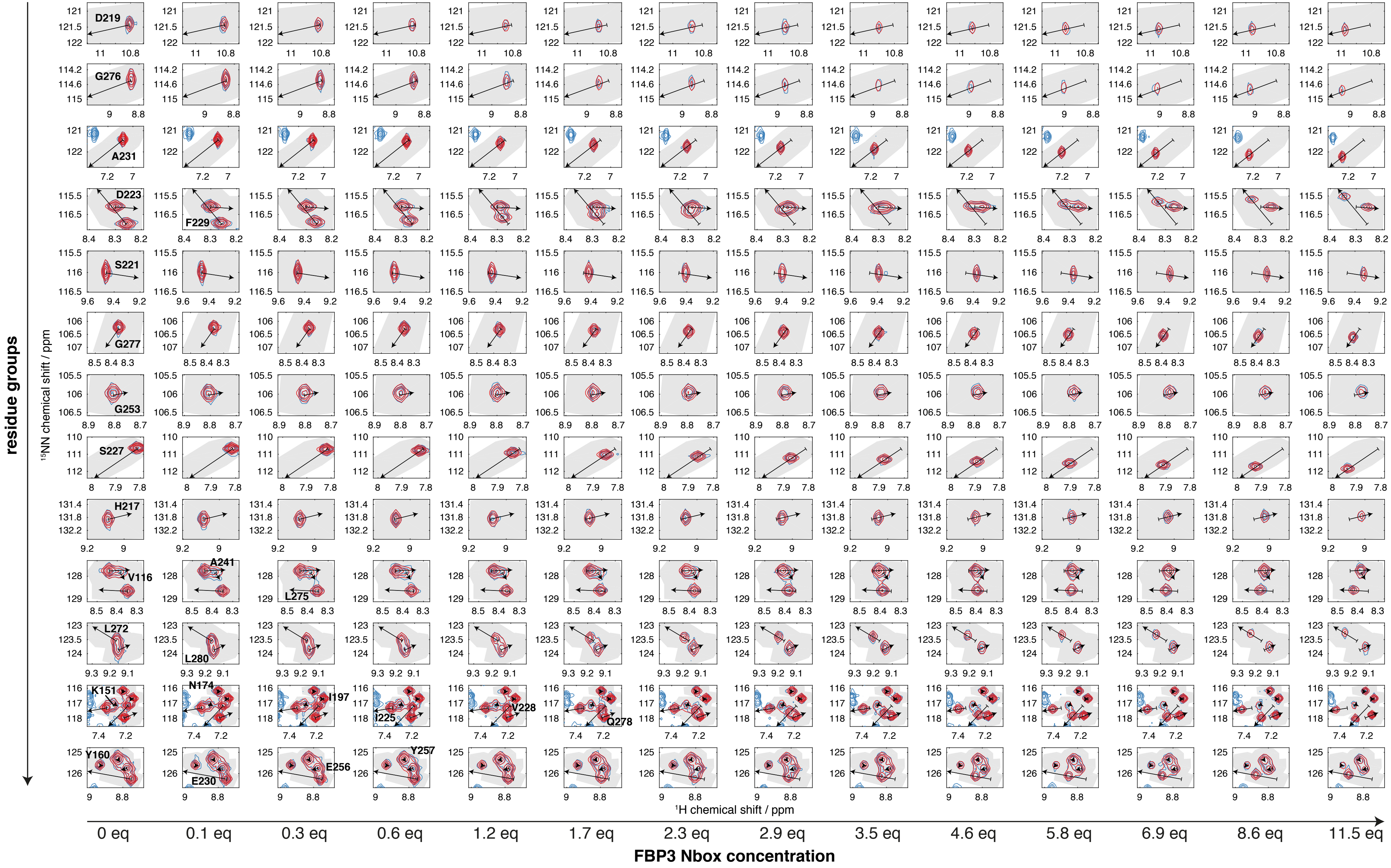

Figure S5. Results of two-dimensional lineshape analysis for the titration of FIR RRM1-RRM2 with FBP3 Nbox. Blue, observed; red, fitted. Shaded areas indicate the selected regions of interest (ROIs). The chemical shifts of free and bound states determined by the fitting procedure are marked by the tail and head of the arrows.

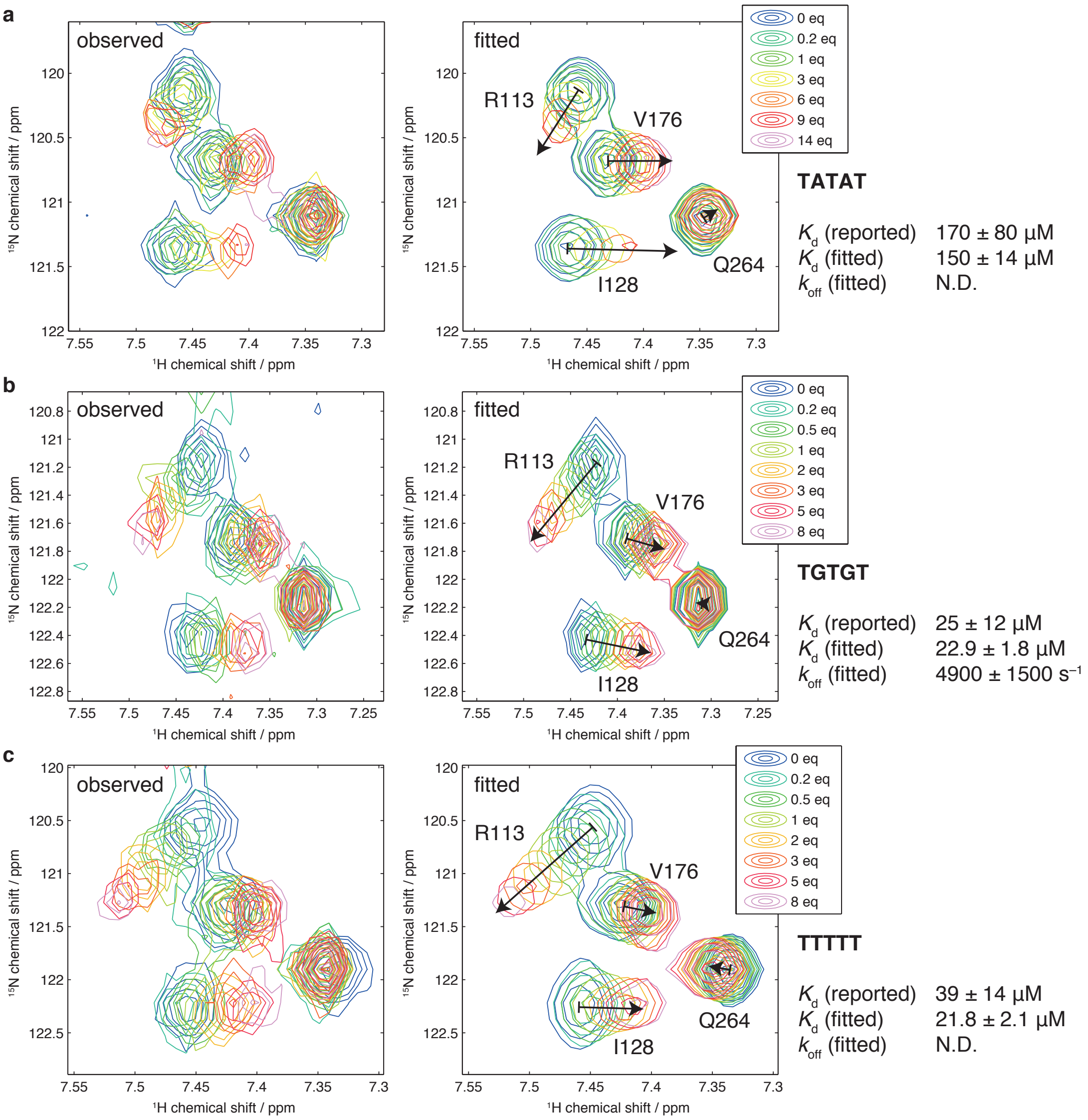

Figure S6. Results of two-dimensional lineshape analysis for titrations of FIR RRM1-RRM2 with the oligonucleotides (**a**) TATAT, (**b**) TGTGT, and (**c**) TTTTT. The chemical shifts of free and bound states determined by the fitting procedure are marked by the tail and head of the arrows shown in the fitted spectra.

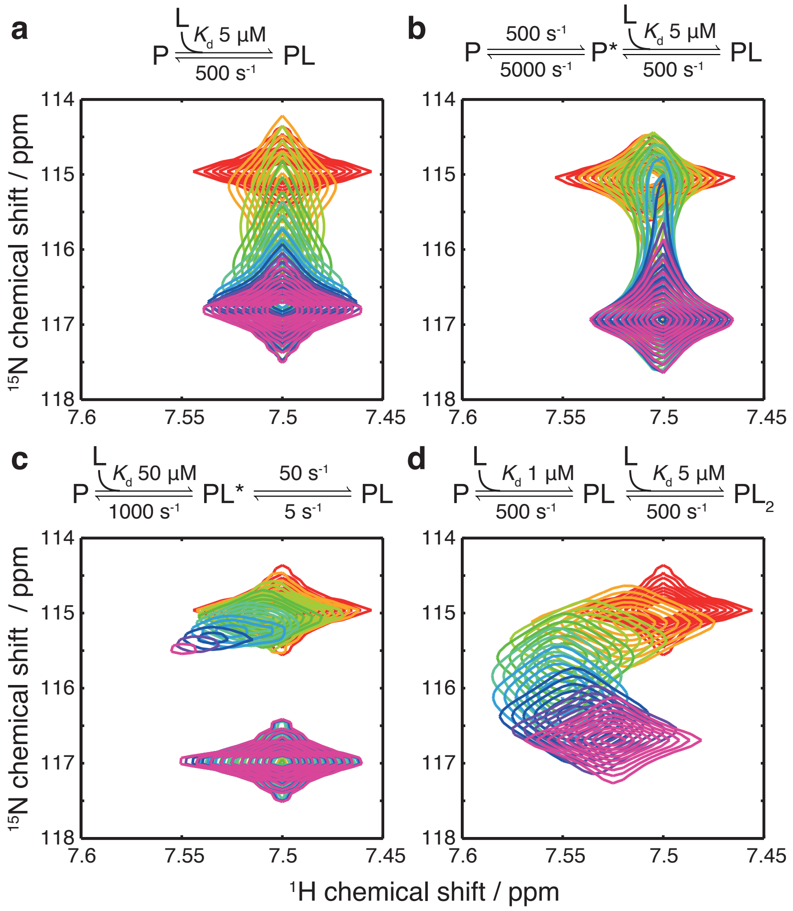

Figure S7. Simulation of a variety of binding mechanisms to illustrate the potential for 2D NMR measurements to discriminate between different mechanistic scenarios: (**a**) simple two-state association; (**b**) conformational selection; (**c**) induced fit; and (**d**) two sequential association reactions. ^{1}H,^{15}N-HSQC spectra are shown simulated at 700 MHz, 50 µM protein concentration, 0 µM (red) to 100 µM (purple) ligand concentrations. Dissociation constants and rate constants are indicated on the reaction schemes above each panel.

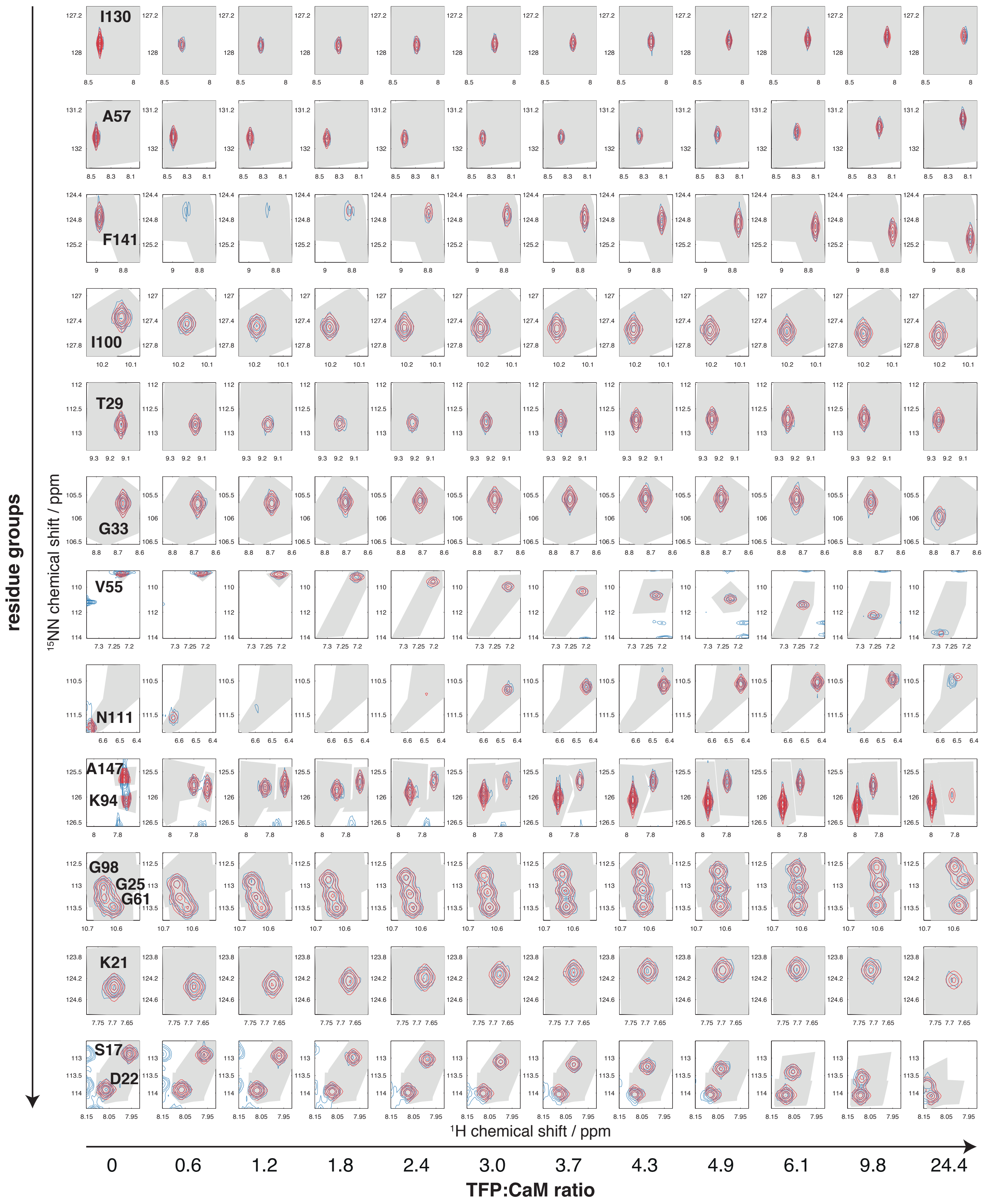

Figure S8. Results of two-dimensional lineshape analysis for the titration of Ca^{2+}-CaM with TFP. Blue, observed; red, fitted. Shaded areas indicate the selected regions of interest (ROIs).

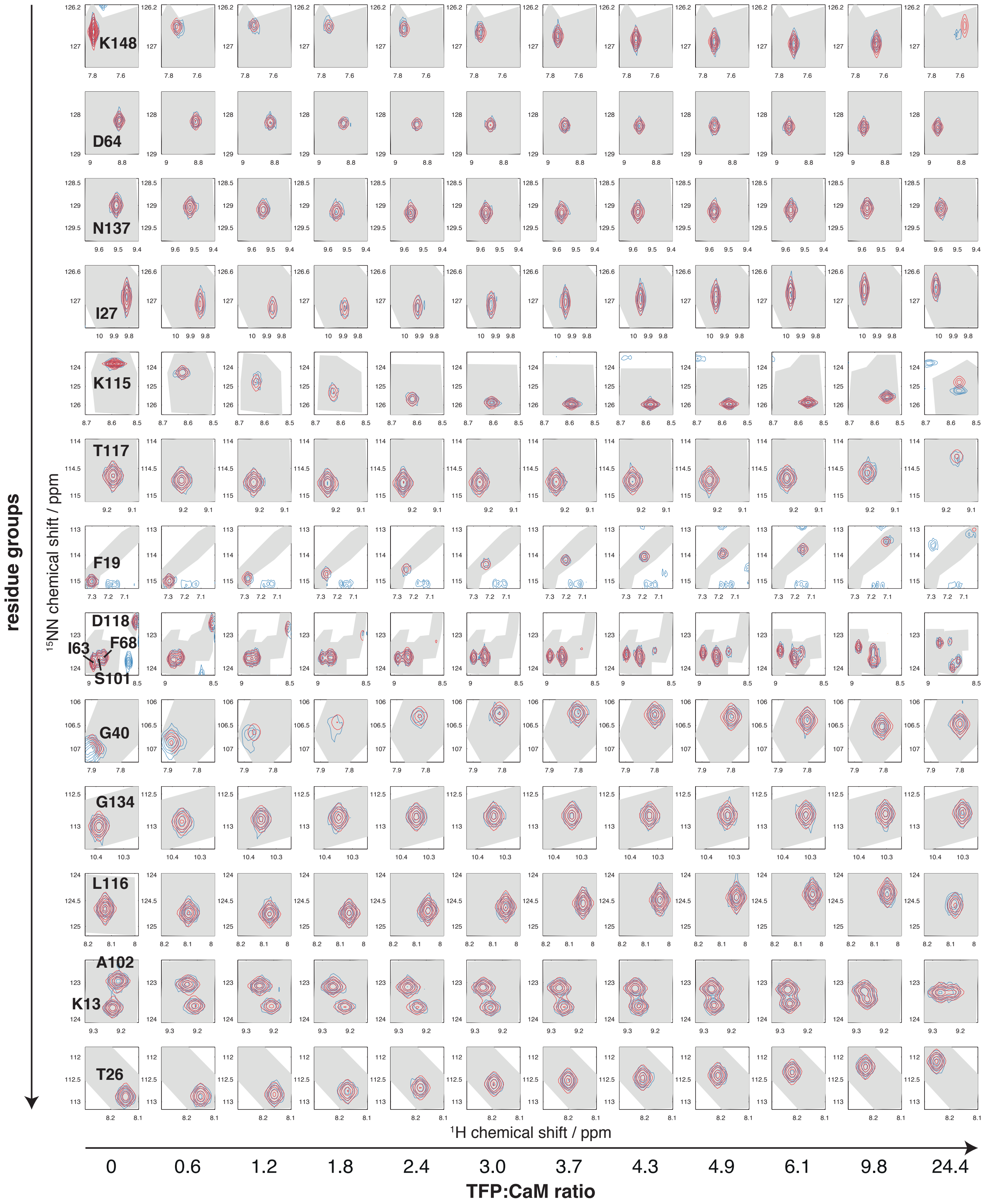

Figure S8 (cont.)

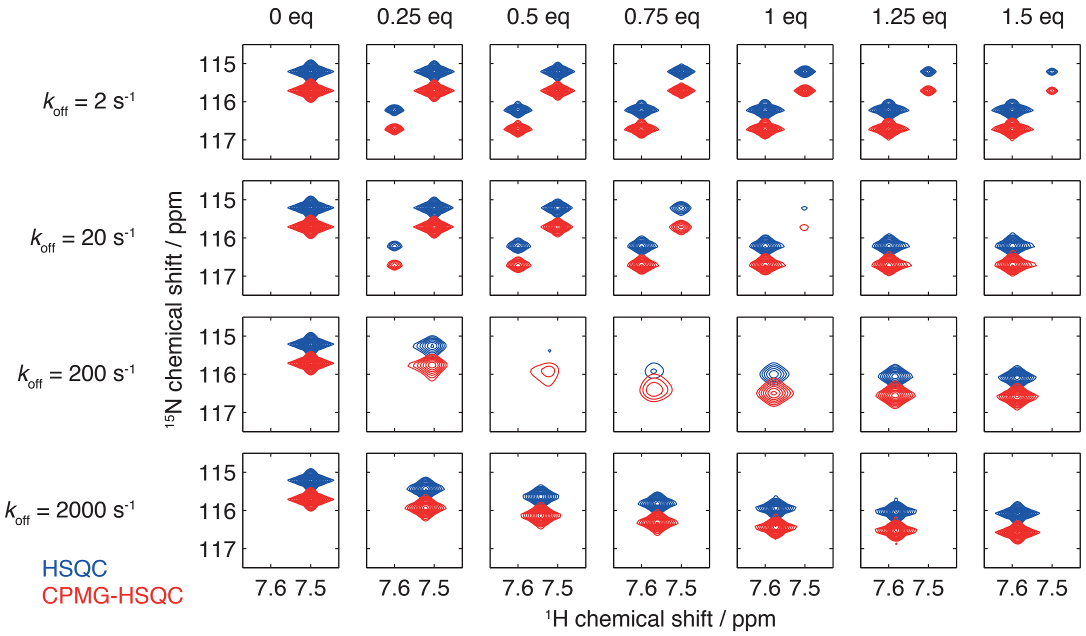

Figure S9. Comparison of HSQC (blue) and CPMG-HSQC (red) pulse sequences for a simulated two-state binding reaction (50 μM protein concentration, 5 μM K_d, 700 MHz ^{1}H Larmor frequency) with dissociation rates and ligand concentrations as indicated. Contour levels are fixed across all spectra.
